# Location and Time Aware Multitask Allocation in Mobile Crowd-Sensing Based on Genetic Algorithm

**DOI:** 10.3390/s22083013

**Published:** 2022-04-14

**Authors:** Aridegbe A. Ipaye, Zhigang Chen, Muhammad Asim, Samia Allaoua Chelloug, Lin Guo, Ali M. A. Ibrahim, Ahmed A. Abd El-Latif

**Affiliations:** 1 School of Computer Science and Engineering, Central South University, Changsha 410083, China; aa.ipaye@hotmail.com (A.A.I.); asimpk@csu.edu.cn (M.A.); guolincsu@csu.edu.cn (L.G.); alimaher_912@hotmail.com (A.M.A.I.); 2 Department of Information Technology, College of Computer and Information Sciences, Princess Nourah bint Abdulrahman University, P.O. Box 84428, Riyadh 11671, Saudi Arabia; 3 EIAS Data Science Lab, College of Computer and Information Sciences, Prince Sultan University, Riyadh 11586, Saudi Arabia; 4 Department of Mathematics and Computer Science, Faculty of Science, Menoufia University, Shebin El-Koom 32511, Egypt

**Keywords:** crowd-sensing, genetic algorithm, incentive mechanism, multitask allocation, time-sensitive

## Abstract

Mobile crowd-sensing (MCS) is a well-known paradigm used for obtaining sensed data by using sensors found in smart devices. With the rise of more sensing tasks and workers in the MCS system, it is now essential to design an efficient approach for task allocation. Moreover, to ensure the completion of the tasks, it is necessary to incentivise the workers by rewarding them for participating in performing the sensing tasks. In this paper, we aim to assist workers in selecting multiple tasks while considering the time constraint of the worker and the requirements of the task. Furthermore, a pricing mechanism is adopted to determine each task budget, which is then used to determine the payment for the workers based on their willingness factor. This paper proves that the task-allocation is a non-deterministic polynomial (NP)-complete problem, which is difficult to solve by conventional optimization techniques. A worker multitask allocation-genetic algorithm (WMTA-GA) is proposed to solve this problem to maximize the workers welfare. Finally, theoretical analysis demonstrates the effectiveness of the proposed WMTA-GA. We observed that it performs better than the state-of-the-art algorithms in terms of average performance, workers welfare, and the number of assigned tasks.

## 1. Introduction

Recently, the mobile crowd-sensing (MCS) paradigm has gained attention from many researchers. The main advantage of MCS is that the deployment of several static sensors over a large geographical region is reduced and replaced by willing users with the required sensing equipment in their smart devices [1]. Furthermore, due to its efficiency, MCS is used in many applications, such as environmental monitoring, prediction of traffic congestion, and map updating [2].

The mobile crowd-sensing mechanism consists of three components: the service requester, platform and workers. The entity that requires the results of the sensing tasks is known as the sensing task requester. Participants are smartphone employees compensated for their contributions and have the requisite sensors to execute the sensing duties. The platform’s purpose is to broadcast task requirements to available participants, such as sensing time, reward, and location, to recruit the best participants for each job, and ultimately to collect and deliver the sensed data to the platform [3,4].

In practice, many MCS systems require that workers consider the time-sensitivity of the tasks. Aware of this requirement, the research community has studied task allocation for the MCS system. Several works focus on task allocation [5,6,7,8]; however, they mainly concentrate on single task assignment, which has been an issue since the time that the worker would have taken to perform multiple tasks is wasted on waiting for the next task allocation from the platform after finishing the previous one.

In actuality, the majority of qualified workers located in the sensing task’s geographical region can only do the tasks during their off-work hours (e.g., half an hour lunch break at work or when returning home after school). Furthermore, each task can be assigned to several workers with the caveat that each worker’s assigned tasks must be completed before the worker’s working time is up. As a result, while scheduling the completion order of assigned tasks, each worker must consider their working time. Each task should be given to workers who arrive at the task’s location and have adequate working time to complete the assignment. As a result, the MCS system’s supremacy is ensured by performing the sensing tasks within the stipulated time range.

Another critical factor to be considered is the payment of the worker. These workers use their valuable time and resources to complete the sensing task and, therefore, they need to be rewarded to encourage them. Unfortunately, in [9,10,11], the researchers focused mainly on improving the utility of the platform and neglected the profit of the workers.

This paper addresses task allocation for the workers and maximization of the total workers welfare considering the strict time constraint. We propose a multitask allocation scheme for time-sensitive tasks based on a genetic algorithm to answer this question.

The main contributions of our paper are summarized as follows:

- We presented a task allocation approach in the MCS system under a given set of allocated task budgets and workers’ available time, which is formulated as a workers welfare maximization problem.
- We proposed an algorithm called Worker Multi-task Allocation (WMTA-GA) that enables workers to select the sensing tasks that are convenient for them and will maximize their profit.
- Simulation shows that WMTA-GA obtains better performance, generates adequate workers welfare, and allocates more tasks to the workers than the existing approaches.

The remainder of the paper is organized as follows. The related works are discussed in Section 2, and the system model together with the problem statement are presented in Section 3. We concentrate on the pricing mechanism and look into the proposed algorithm in Section 4 and Section 5, respectively. Section 6 focuses on the experimental design and discussion of the results. Finally, the paper is concluded in  Section 7.

## 2. Related Work

### 2.1. Task Allocation

Task allocation is an essential issue in the MCS system as it determines the performance of the system. Therefore, many studies in task allocation approaches have been investigated. In [12], the authors aim to solve the task-allocation problem by employing a bilateral preference matching game between the service requester and the workers in the MCS system to satisfy both the service requester and the workers. In some cases, a sensing task needs to be allocated to more than one worker to increase the workers’ utility. The authors in [5] tackle this problem by designing an approach that uses both a greedy and an equilibrium-based method. In [13], a new protocol for assigning tasks was introduced to measure the workers’ utility when performing sensing tasks in a crowd-sensing opportunistic network [2]. In [14], the purpose was to use an analytical hierarchy process to select workers from the user set. The worker had the option of accepting the sensing task, and if it chose to do so, then its updated reputation was to be determined based on its performance. There was also an issue of unbalanced task allocation that needed to be resolved to improve the performance of MCS. The purpose of [15] was to solve the problem of online user recruitment with consideration of the budget and time constraints.

However, for the allocation of time sensitive tasks, the above task allocation approaches will be ineffective. Therefore, the research in [16,17,18,19,20] focused on improving the MCS system’s reliability by ensuring that the sensing task is completed before the specified deadline. In [21], the authors proposed an approach such that the workers would spend less time performing the sensing task. This aim was achieved by partitioning each task into groups, and a travelling path for each worker was planned. The authors in [3,22,23] noted that most task-allocation research focused on assigning single tasks to workers. However, these workers usually had the capacity to complete multiple tasks. Due to this reason, a mechanism was introduced for the multitask allocation to maximize the platforms’ utility. In [24], the users’ preference based on content and context information was used to develop a worker-recruitment mechanism. The authors in [25] studied user preference and the effect it had on task assignment and workers’ long-term utility. The authors in [7] studied a destination-aware task-allocation strategy to maximize the number of completed tasks and perform tasks before the deadline. In [26], the authors determined which sensing task to complete according to the incomplete task-popularity statistics information. Some papers focused on collecting the sensed data from the workers and delivering it to the platform. In [27], a mechanism was introduced to transmit sensed data to the data requester according to the time constraint. In [28], the researchers aimed to collect sensed data from workers that have high coverage properties of MCS fulfilled. The authors in [29] proposed a data-collection path-planning scheme that employed either cellular transmission or an opportunistic network.S. Akter et al. [30] investigated task allocation in time-sensitive MCS based on A-DQN. The task allocation problem was considered a travelling salesman problem, and the aim was to minimize the total cost of completing the tasks subject to time constraints.

### 2.2. Incentive Mechanism

It is important that the workers assigned to the sensing tasks are compensated, as these workers consume their computational resources and time, and are at risk of private information leakage. Therefore, an incentive mechanism must be presented to encourage workers to participate in the MCS system [31,32,33,34]. In [35], a truthful incentive approach was introduced, which used the workers’ sensing performance and reputation to determine the payment they would receive after completing the allocated tasks. In [36,37], the tasks were allocated such that the social welfare was maximized to ensure that there is fairness in the system. In [38], the semi-Markov model was used to obtain the positions of the workers, which was then used to determine workers with the least distance and bidding price. The authors in [39] updated the reward of each task in each iteration to take advantage of the workers’ interest in a particular task. However, this research on incentive mechanisms neglected the fact that maximizing the combined utility of the workers will encourage them to participate in time-sensitive tasks in MCS and increase the chances that they will be optimistic about performing the future time-sensitive tasks as well.

### 2.3. Summary

According to the research mentioned above, most authors failed to consider maximizing the workers welfare for time-sensitive tasks and its benefit for MCS. For instance, in [30], the authors proposed A-DQN to determine the task allocation subject to time constraints. However, they aimed to minimize the total reward given to the users selected to perform the sensing task. Therefore, by considering the task allocation subject to time constraints of the task and workers, this paper aims to maximize the total worker welfare by solving the (WMTA-GA) multitask allocation problem.

## 3. System Model and Problem Statement

In this section, the system model of the MCS system followed by the problem statement are presented. Table 1 shows the main notations used in this paper.

### 3.1. System Model

**Figure 1:**
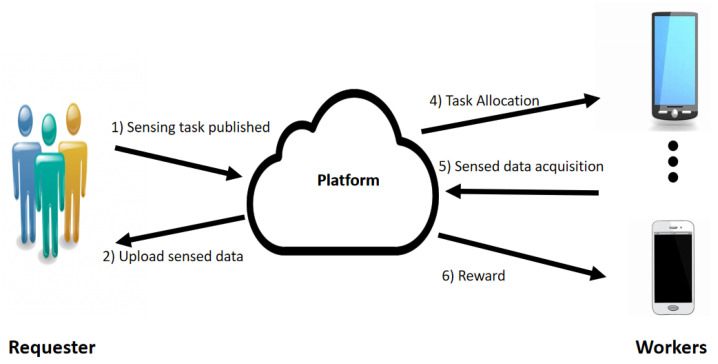
MCS System Model.

Figure 1 depicts the MCS system model and the following definitions describe the components of the model:

**Definition 1** (Requester)**.** *The requester is an entity; which can be an individual or a company, that requires certain sensing tasks $T=\{t_{1},t_{2},t_{3},\cdots ,t_{n}\}$ to be performed. The requester communicates with the platform about its required sensing tasks and provides a budget for the set of tasks to be performed.*

**Definition 2** (Task)**.** *Each task $t_{j}\in T$ is located in a specific geographical position $p_{j}$ . The profile of task $t_{j}$ is denoted by $Pr_{j}=\left\{p_{j},bt_{j},te_{j},ts_{j},will_{j}\right\}$ which consists of budget $bt_{j}$ , sensing-duration $\left(te_{j},ts_{j}\right)$ , and willing threshold $will_{j}$ .*

**Definition 3** (Platform)**.** *The main responsibility of the platform is to assign tasks to the workers having the necessary sensor component and who are willing to perform the specific task. When a worker $w_{i}$ has available working time, it informs the platform, and the worker will select the set of tasks that they can perform. All the sensed data are then collected by the platform and sent to the corresponding requester.*

**Definition 4** (Worker)**.** *The worker $w_{i}\in W$ is a smart-device user with enough energy that participates in performing sensing tasks. A worker can participate in a sensing task only if the task’s sensing duration fits into the worker’s available working time $wt_{i}$ and the worker’s willingness factor for the task is above the threshold. Each worker is associated with a specification for each task $t_{j}$ which is denoted by $SP_{ij}=\left\{p_{i},wt_{i},c_{ij},v_{i}\right\}$ which consists of current position $p_{i}$ , available working time $wt_{i}$ , sensing cost $c_{ij}$ , and velocity $v_{i}$ . The goal of workers is to select tasks that will maximize their utility. The worker $w_{i}$ then includes these selected tasks to its set of interested tasks $T_{t}^{w_{i}}=\left\{t_{1},t_{2},t_{3},\cdots ,t_{f_{j}}\right\}$ . After selecting these tasks, the worker sends to the platform its profile $Pr_{i}=\left\{T_{t}^{w_{i}},SP_{iT_{t}^{w_{i}}}\right\}$ .*

### 3.2. Problem Statement

From the description of the system model, the task-allocation problem is formulated as follows:

(1) $$\max\sum_{i=1}^{n}\left(R_{i}\cdot a_{i}-c_{i}\right)$$

s.t.

(2) $$\sum_{j=1}^{m}D\left(w_{i},t_{j}\right)\leq v_{i}\cdot wt_{i},\forall t_{j}\in T_{t}^{w_{i}}$$

(3) $$D\left(w_{i},t_{j}\right)\leq v_{i}\cdot \left(te_{j}-ts_{j}\right),t_{j}\in T_{t}^{w_{i}}$$

(4) $$WF_{ij}\geq will_{j},\forall t_{j}\in T$$

(5) $$a_{ij}=1\;\mathrm{or}\;a_{ij}=0,\forall w_{i}\in W,\forall t_{j}\in T$$

(6) $$\sum_{i=1}^{n}a_{ij}\leq 1,\forall t_{j}\in T$$

where $R_{i}$ denotes the payment given to $w_{i}$ , the distance between the worker’s location and the location of task $t_{j}\in T_{t}^{w_{i}}$ is denoted by $D(w_{i},t_{j},T_{t}^{w_{i}})$ , velocity is indicated by *v* and $a_{ij}$ represents the task-assignment: if task $t_{j}$ has been assigned to worker $w_{i}$ then $a_{ij}=1$ otherwise $a_{ij}=0$ .

Equation (2) indicates that the time that the worker $w_{i}$ takes to move from their current location to the last task in $T_{t}^{w_{i}}$ should not exceed the working time $wt_{i}$ . This constraint is to ensure that the worker can perform all the allocated tasks it has accepted from the platform within its working time. Constraint Equation (3) ensures that each task is completed within the sensing duration, Equation (4) ensures workers with a high willingness factor are selected to perform task $t_{j}$ , and Equation (5) ensures that each task is allocated to only one suitable worker.

The willingness-factor $WF_{ij}$ predicts the performance of workers for the assigned sensing tasks. The $WF_{ij}$ is obtained by considering worker $w_{i}$ response-time $Wrt_{ij}$ (i.e., the time the worker begins a sensing task) and the residual power of the workers’ smart device $p_{i}^{r}$ . For example, when the worker’s commencing time for a task is the same as the task’s start time, then the response time of the worker is 0. This reflects positively on the workers’ willingness to perform the task, and in this scenario, the willingness-factor is 1. From the above analysis, the willingness-factor is measured as follows:

$$WF_{ij}=\left\{\begin{array}{cc} \min\left(\left[\frac{P_{i}^{r}}{P_{i}^{t}}.\frac{\left(\sum_{i=1}^{n}\frac{Wrt_{ij}}{n}\right)}{Wrt_{ij}}\right],1\right), & Wrt_{ij}\neq 0\\ 1, & Wrt_{ij}=0 \end{array}\right.$$

The willingness factor is then normalized as follows,

(7) $$NWF_{ij}=\left(WF_{ij}\right)/\left(\sum_{j=1}^{m}WF_{ij}\right)$$

and worker $w_{i}$ is then rewarded with the following:

(8) $$R_{i}=\left(\sum_{j=1}^{m}NWF_{ij}\right)\cdot bt_{j}$$

where $bt_{j}$ is the budget allocated to task $t_{j}$ . The calculation of the task budget is discussed in Section 4.

**Theorem 1.**  *The multitask allocation problem is NP-complete.*

**Proof.**  The multitask allocation problem is a combinatorial optimization problem that has a large solution space. We assume that there is *Q* number of tasks and *s* number of workers, and each worker can perform *Q* number of tasks. Since each worker can perform $2^{Q}$ of assigning tasks and for all *s* number of workers, there will be $2^{sQ}$ assigning method. In the worst case, there will be $2^{sQ}\cdot Q!$ assignment method if each worker develops *Q*! ways of completing the tasks, increasing the solution space which implies that the proposed multitask problem is NP-complete.    □

## 4. Pricing Mechanism

In this section, a pricing mechanism is introduced to determine the budget allocation for each task.

The task budgets are allocated to each task according to the task demand $TD_{j}$ which is a reflection of how important a task is in the MCS system. The budget is obtained by the weighted combination of the task-popularity $W_{T_{i}}$ and the task-duration $t_{d_{j}}$ which is defined by the following

(9) $$t_{d_{j}}=t_{e_{j}}-t_{s_{j}}$$

The task popularity reflects how frequently workers visit a certain location and is indicated by the predicted number of workers in a given task location. The task demand for task $t_{j}$ is determined as follows:

(10) $$TD_{j}=\left(w_{1}*W_{T_{i}}\right)+\left(w_{2}*t_{d_{j}}\right)$$

where $w_{1}$ and $w_{2}$ are the weights satisfying ( $w_{1}$ + $w_{2}$ = 1), the values which are determined by the MCS platform. However, in this paper, we assume that the sensing duration is more important than task popularity, and therefore we set the weights as $w_{1}=0.2$ and $w_{2}=0.8$ .

The task demand is then normalized as follows,

(11) $$NTD_{j}=\left(TD_{j}\right)/\left(\sum_{j=1}^{m}TD_{j}\right)$$

and therefore given the total budget *B* of all tasks, the budget allocated to task $t_{j}$ is given by,

(12) $$bt_{j}=\left(NTD_{j}\right).B$$

## 5. Multi-Task Allocation Algorithm

From the previous section, it was concluded that the task allocation solution space was too large, and traditional combinatorial optimization algorithms will be inefficient in handling the multitask allocation problem. In this section, we introduce the genetic algorithm (GA) [40] to solve the multitask allocation problem. GA has been applied to address various optimization problems in various research fields [41,42,43,44].

### Genetic Algorithm for Multitask Allocation

In order to solve the studied problem, WMTA-GA adopted GA for multitask allocation as presented in Algorithm 1. First, the population is initialized with *K* chromosomes (line 1). In each iteration, the best chromosome is selected based on fitness (lines 2–9). The fitness presented in line 4 represents the objective problem that is used to evaluate each chromosome’s performance. The current best chromosome is updated in line 5, and the selection operator, crossover and mutation operator, and re-establish operator are introduced in (lines 6–8), respectively. The while loop continues until all the workers have been assigned to a specified set of tasks. Finally, the global best chromosome is updated in line 11. The initial population is generated from Algorithm 2, which uses the greedy algorithm.  The re-establishing operator is presented in Algorithm 3.
**Algorithm 1** WMTA-GA.

**Input:** 
Worker set *W* and Task set *T***Output:** 
Worker set $S_{W}$ assigned to the task $t_{j},\forall t_{j}\in T$
  1:
$Pop_{size},max_{G},G$
  2:Initialize population initial *G* with *K* chromosomes  3:**while** $G\leq max_{G}$ **do**  4:      Evaluate the fitness of all chromosomes in $Pop_{G}$
  5:      Update the current best chromosome  6:      
$Pop_{G}^{\prime }$ ← Selector operator on $Pop_{G}$
  7:      
$Pop_{G}^{\prime \prime }$ ← Crossover and mutation operator on $Pop_{G}^{\prime }$ to produce update *N* chromosomes  8:      
$Pop_{G+1}$ ← Re-establish operator on $Pop_{G}^{\prime \prime }$
  9:      *G*← G+1
 10:**end while** 11:**return** best chromosome (task-worker allocation)

**Algorithm 2** Initial population.

**Input:** 
Worker set *W* and Task set *T*
**Output:** 
Chromosome C (initial task-worker allocation)  1:Candidate worker set WS ←*W*  2:Unassigned task set TS ←*T*  3:**while** WS ≥ 0 **do**  4:    Randomly select a worker *w*∈ WS
  5:    Set counter q=0
  6:    **while** q≤ number of tasks **do**  7:        Randomly select a task $t_{j}$ ∈ TS
  8:        *C*← append $t_{j}$ to the end of the genetic segment allocated for *w*  9:        **if** *C* is a supported chromosome **then** 10:        
TS ← $TS-t_{j}$
 11:        **end if** 12:        *q*← q+1
 13:    **end while** 14:    
WS ← WS−w
 15:**end while**

**Algorithm 3** Re-establishing Operator.

**Input:** 
A unsupported chromosome C
**Output:** 
A unsupported chromosome $C_{n}$
  1:**Case 1: Unsupported genetic segment**  2:**for** genetic segment C(w) ∈ *C* **do**  3:**if** gene g(i) ∈ C(w) violates constraints Equations (2)–(4)  4:*C*(*w*)← select subset of genes in the genetic segment that satisfy the aforementioned constraints  5:**end if**  6:**end for**  7:**Case 2: Repeated task allocation**  8:**for** each task $t_{j}$ **do**  9:
RGS ← all genetic segments containing the same task $t_{j}$
 10:
GSB ← the genetic segment ∈ RGS with the maximum fitness 11:**for** the remaining genetic segment in RGS **do** 12:Remove task $t_{j}$
 13:**end for** 14:**end for** 15:**for** genetic segment C(w) ∈*C* **do** 16:Update worker *w* position and remaining working time 17:
$T_{a}$ ← the tasks that *w* can perform with remaining available time 18:**while** $\;\mathrm{do}\;T_{a}$ ≥ 0 **do** 19:    Randomly select a task $t_{j}^{\prime }$ from $T_{a}$
 20:    
$C^{\prime }\left(w\right)$ ← $C\left(w\right)\cap t_{j}^{\prime }$
 21:    **if** $C^{\prime }\left(w\right)$ is a supported chromosome **then** 22:    
C(w) ← $C\left(w\right)\cap C^{\prime }\left(w\right)$
 23:    **end if** 24:    
$T_{a}$ ← $T_{a}-t_{j}^{\prime }$
 25:**end while** 26:**end for**

1. Chromosome Representation and Fitness Evaluation
  The main aim of WMTA-GA is to find a solution that indicates task–worker allocation and task completion order. Since the number of chromosome solutions is uncertain, we use a matrix structure to represent them. Each chromosome consists of *b* genetic segments representing the registered workers. The genetic segments are identified using the index of their position in the chromosome. Each genetic segment is divided into genes that represent the tasks assigned to the worker. According to the constraints Equations (2)–(6), we classify the chromosomes into supported chromosomes meaning that the multitask allocation satisfies the constraints and unsupported chromosomes when the constraints are not satisfied.
  Since each chromosome represents all the workers and their assigned tasks, the whole population represents the set of workers and their assigned tasks.The fitness function was applied to the chromosomes in a population to evaluate the quality of the population. Since the objective function $R_{i}=\left(\sum_{j=1}^{m}NWF_{ij}\right).\;bt_{j}$ is to maximize the workers welfare, then it is used as an indicator of each chromosome’s fitness level (i.e., the amount of workers welfare generated by a chromosome is directly related to its fitness level). Therefore, the fitness function of a chromosome $C_{t}$ is calculated as:
(13) $$Fit\left(C_{t}\right)=\sum_{i=1}^{n}R_{i}.a_{i}-c_{i}$$
  This implies that for a population in a given generation defined as G = {C1, C2, C3, …, Cl}, the task assignment strategy of chromosome $C_{t}$ (1≤t≤l) is the row vector $A_{h}=\left\{a_{1},a_{2},a_{3}\cdots ,a_{p}\right\}$ , where $a_{i}$ (1≤i≤p) can be either “1” if the task is allocated to the worker, or otherwise $a_{i}$ is “0”.
2. Selection Operator
  The selector operator aims to allow the chromosomes with the higher fitness level to proceed to the next generation while taking into consideration the population diversity; however, chromosomes with lower fitness values can contain acceptable genetic segment properties. To guarantee that top-quality chromosomes are accepted into the next generation, we firstly select elitist chromosomes to ensure that chromosomes with high fitness levels do not disappear. To obtain this type of chromosome, we arranged the parent population chromosomes in descending order of fitness, and randomly picked out a specified number of chromosomes (e.g., first to fifth) and passed it on to the next generation. Afterward, tournament selection [3] is applied to the remaining chromosomes. In each generation, a certain number of chromosomes are compared and the chromosome with the highest fitness level proceeds to the next generation. This continues until the required amount is obtained.
3. Crossover and Mutation Operator
  In the crossover operation, a new generation of sophisticated chromosomes is generated by recombining the parent chromosomes through crossover operation. Initially, two-parent chromosomes from the elite set of chromosomes and a single chromosome from the remaining chromosomes are selected. Then the fitness value of the genetic segments of the chromosomes (elite and normal chromosomes that are to be combined by crossover operation) are compared using their fitness level, and the superior genetic segment is passed to the new chromosome. In the process of performing this operation, the entire population is evolved and the diversity of the population is improved.
  It is important to realize that the new chromosome generated from the crossover operation might not follow the constraint conditions and therefore become an unsupported chromosome. To avoid local optimum and enhance the population diversity, the mutation operation is applied to the chromosome.
  For the chromosome mutation to occur, we select two genes from two genetic segments in the same chromosome and swapped them to generate mutated chromosomes. However, during the mutation process, the status of some chromosomes may change and become unsupported.
4. Re-establishing Operator
  After the crossover and mutation operation, some supported chromosomes may convert to unsupported chromosomes and for that reason, we introduce a re-establishing operator. Initially, we confirm whether each genetic segment satisfies all the constraints. If a constraint is violated, then we select a subset of genes in the corresponding genetic segment that satisfies the constraints. Secondly, if a task is allocated more than once in a particular chromosome, we use the idea of the survival of the fittest to determine which of the tasks is to be retained. Finally, we consider the fact that some workers may have remaining time to perform some unallocated sensing tasks and determine which chromosome should be selected for this task such that the constraint remains satisfied.

## 6. Experimental Results

### 6.1. Experimental Settings

In this section, we introduce the experimental design used to analyze the proposed WMTA-GA algorithm. In the experiment, we assumed that both the number of sensing tasks and the number of workers range from 60 to 200. The maximum number of iterations (i.e., the maximum generation $max_{G}$ ) and the population size is set to 150 and 20, respectively. The worker’s working time ranges from 1 to 50 (min), and the response time of the task ranges from 1 to 20 (min). The sensing area is divided into 100 locations, and the position of both the worker and task are randomly selected from one of the locations. We assume that the velocity of the worker is 50 km/h and the total budget allocated to the platform is 15,000. The willing threshold for each task is randomly selected from the range [0.1 to 0.7]. The sensing cost of the workers is within the range [100, 400]. The experiment was conducted using MatLab 2016a.

### 6.2. Performance Metric

We evaluate the approaches’ performances by studying how various parameters (number of tasks and number of workers) affect the following metrics:

1. Workers Welfare: This metric is an indicator of how well an algorithm performs based on fulfilling the condition of maximizing all the workers’ profit. The higher this profit, the better the performance of the algorithm.
2. Number of Allocated Tasks: This indicates how many tasks are allocated to the selected workers. When the number of allocated tasks is high, it shows that the large number of workers can complete the tasks and this improves the reliability of the platform.
3. Average Performance w.r.t Varying Number of Tasks: The average worker’s welfare subject to the number of tasks in each algorithm is evaluated. This ratio assists in evaluating the performance of the algorithms.
4. Average Performance w.r.t Varying Number of Workers: The performance of each algorithm is compared by evaluating each of their average workers welfare with respect to the number of workers. A high ratio indicates a high-performing algorithm.

### 6.3. Baseline Approaches

1. Greedy Worker Payoff-based Task Allocation (GWP): In [17], the GWP approach was used to greedily select tasks with the highest reward in each iteration. We modified this algorithm to maximize the workers welfare, whereby each worker selects the tasks with the biggest reward and these workers are assigned to their selected tasks if the worker–task allocation satisfies the validity conditions. If the conditions are satisfied, then we exchange the location of the worker with the location of the current task, therefore changing the new initial location of the worker. This process continues until each worker’s working time is finished.
2. Greedy Worker Reward-Distance Ratio-GA (GWR-GA): The second benchmark algorithm is the modified greedy payoff/distance-based task selection algorithm (G.P.D.A.) [17]. This approach has been modified to maximize the workers welfare by employing GA tasks with a high payoff/distance ratio to be assigned to workers, and GA is employed to obtain an optimal chromosome (i.e., worker–task allocation).

### 6.4. Result Analysis

In this section, the results obtained from WMTA-GA, GWR-GA and GWP are interpreted and analyzed.

**Figure 2:**
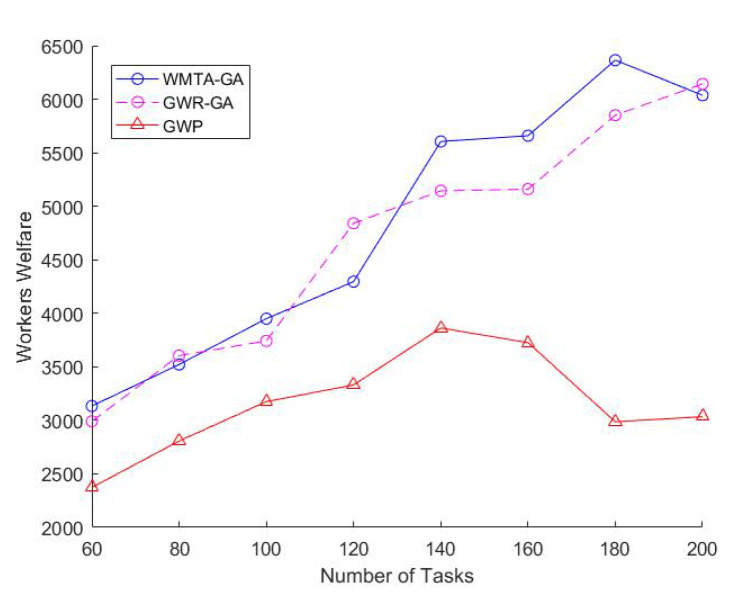
Impact of Varying Number of Tasks on Worker Welfare.

**Figure 3:**
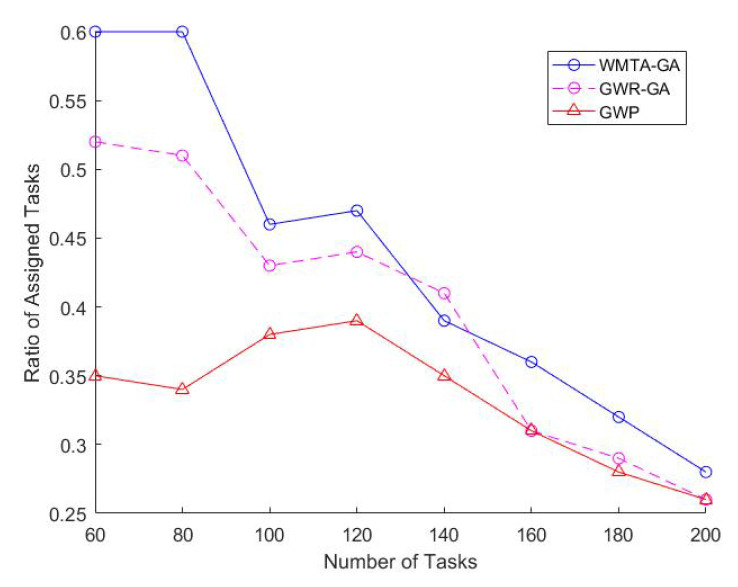
Impact of Varying Number of Tasks on Ratio of Assigned Tasks.

**Figure 4:**
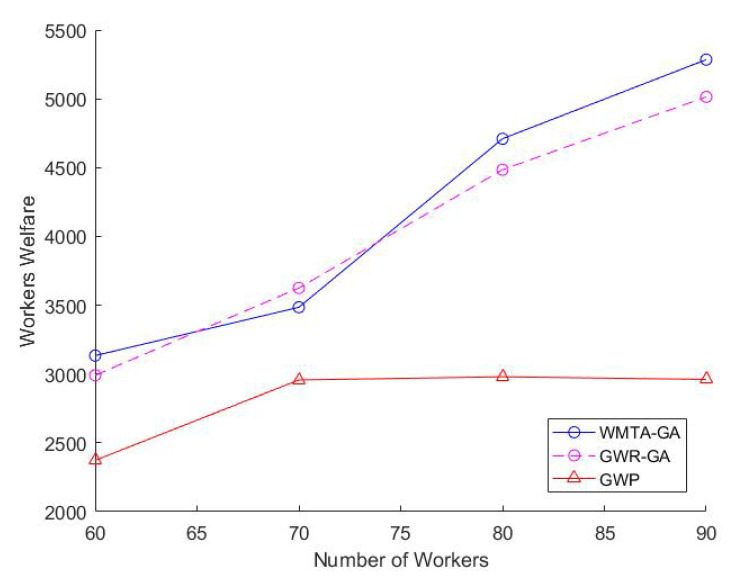
Impact of Varying Number of Workers on Worker Welfare.

**Figure 5:**
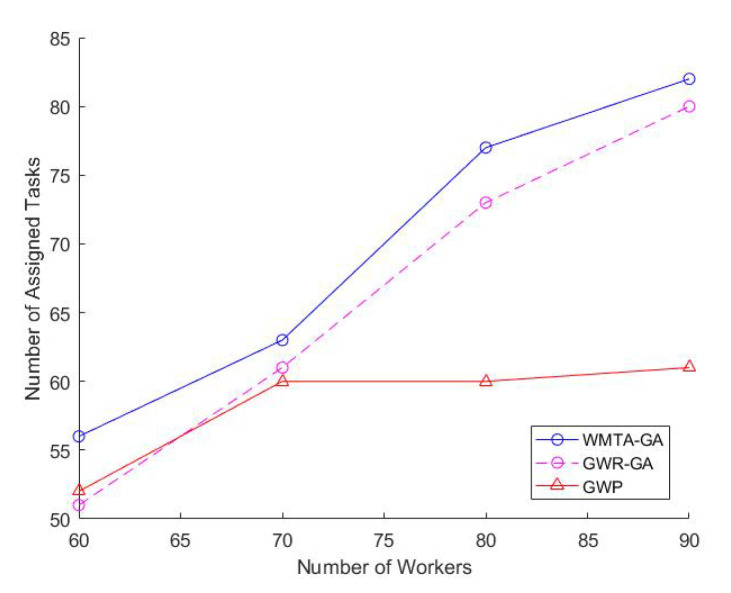
Impact of Varying Number of Workers on Number of Assigned Tasks.

- Impact of Varying the Number of Tasks
  In Figure 2, we observe that the number of tasks is directly proportional to the workers welfare and that WMTA-GA outperforms GWR-GA and GWP. For instance, when the number of tasks was 100 and 140, the workers welfare for WMTA-GA was 3950 and 5605, respectively, which is much higher than the welfare obtained by GWR-GA (3742 and 5144) and GWP(3175 and 3863).
  As shown in Figure 3, the ratio of the assigned tasks in WMTA-GA, GWR-GA, and GWP declines significantly with the increase in the number of tasks. However, when the number of tasks is 60 and 200, we see that the WMTA-GA recorded the highest ratio of assigned tasks, which are 0.6 and 0.26, respectively. Table 2 illustrates the performances of the three approaches and indicates that WMTA-GA performs better in comparison to GWP and GWR-GA. This is confirmed by their average utility value where WMTA-GA is 73.24 which is 13% and 68% higher than GWR-GA and GWP, respectively.
- Impact of Varying Number of Workers
  We investigate how varying the number of workers affects worker welfare, the number of task allocations, and the average performance of the workers. We set the range of workers from 60 to 90 and fixed the number of tasks to 200. In Figure 4, we observe that as the number of workers increases, the workers’ welfare increases as well. The workers’ welfare in WMTA-GA steadily increases compared to the other approaches. For example, when the number of workers ranges from 60 to 80, WMTA-GAs’ worker welfare is 3100 and 5300.
  In Figure 5, we see that WMTA-GA provides the highest number of assigned tasks as compared to the other algorithms. When the number of workers is 60 and 80, WMTA-GA achieves 56 and 77 number of the assigned tasks.
  The number of the assigned tasks increases as more tasks are successfully allocated, as observed in Figure 5. The WMTA-GA approach had the highest number of assigned tasks compared to other approaches for *n* = 60 to 90, with the highest recorded number of assigned tasks of 84 at n=90 . GWR-GA follows the same trend as WMTA-GA because it achieved a similar result to WMTA-GA. However, GWP had the lowest overall number of assigned tasks and peaked at 68 assigned tasks at n=70 .
  We observed from Table 3 that WMTA-GA has the best average performance as compared to GWR-GA and GWP, which is indicated by their average utility values. WMTA-GA’s average utility value is 17.26% more than GWP, and 1.98% more than GWR-GA. The second-best performing algorithm is GWR-GA, with an average utility value higher than GWP by 15 percent. Compared to WMTA-GA and GWR-GA, the GWP had a minor favourable result, with an average performance of −15% and −13.3% for WMTA-GA and GWR-GA, respectively.

### 6.5. Discussion

In Section 6.3, WMTA-GA achieves the overall best results for the workers welfare, ratio of assigned tasks and number of assigned tasks. The explanation for this is that the workers using the WMTA-GA approach have a higher chance of being correctly assigned to sensing tasks with greater reward due to the re-establishing operator. The function of the re-establishing operator is to convert the unsupported chromosome (i.e., task-worker allocation) to the supported chromosome that fulfils the MCS constraints Equations (2)–(4). For instance, in Figure 2, WMTA-GA recorded high worker welfare as a value of *m* increases, while GWR-GA followed a similar trend but with lower values for the workers welfare. On the other hand, GWP, which uses the greedy algorithm, had the lowest worker welfare throughout the graph. In Figure 3, the ratio of assigned tasks declined for all approaches as the number of tasks increased. This result is due to the resources of the workers in the MCS system being limited, so newly introduced tasks will be less likely to be assigned as the workers will have exhausted their resources from the previous set of tasks. Nevertheless, the WMTA-GA displayed a better ratio of assigned tasks than the remaining approaches due to the assistance of the re-establishing operator.

The advantages of using the re-establishing operator for the various number of workers are presented in Figure 4 and Figure 5. In Figure 4, WMTA-GA accomplished the highest steady increase in worker welfare for the varying number of workers. The stronger performance of WMTA-GA can be attributed to the following reasons; (1) more tasks are being successfully assigned to the workers in the MCS system, and, as a result, the selected workers gain more profit. Due to similar reasons, WMTA-GA in Figure 5 experienced the highest number of assigned tasks for the range *m* = (60 to 90) and (2). The average percent of supported and unsupported chromosomes in each iteration is 30% and 70%, respectively. The unsupported chromosomes are then reduced and converted into supported chromosomes by using the re-establishing operator.

Therefore, we observed that the approaches that do not utilize a re-establishing operator will have lower performance due to the unsupported chromosome or task allocation. Therefore, we can conclude that WMTA-GA is superior to GWR-GA and GWP.

## 7. Conclusions

In this paper, we have studied a multitask allocation in a mobile crowd-sensing environment. The problem is formulated as an optimization problem. We first proved that the problem is NP-complete, which is hard to be solved by traditional methods. Then, we proposed a worker multitask allocation-genetic algorithm (WMTA-GA) to solve this problem. WMTA-GA is used to optimize the workers’ welfare such that the multiple sensing task is allocated to a worker with the condition that the time and willingness constraints are fulfilled. Specifically, these conditions include that the allocated tasks are performed during the working time of the worker, and the worker’s willingness factor for each assigned task is above the tasks’ willingness threshold. For each selected worker, we utilized the task budget and the worker’s willingness factor to determine the payment for the worker. Simulation results show that the proposed WMTA-GA approach outperforms other approaches in terms of average performance, worker’s welfare, and ratio and number of assigned tasks. 

## Figures and Tables

**Table 1:** Model Parameters.

System Model Parameter	
**Worker Parameter**	
*W*	Set of all workers
*n*	Number of workers
$w_{i}$	worker *i*
$c_{i}$	Sensing cost of worker $w_{i}$
$p_{i}$	Current position of worker $w_{i}$
$wt_{i}$	Available working time of worker $w_{i}$
$P_{i}^{t}$	Power level of worker $w_{i}$
$v_{i}$	Velocity of worker $w_{i}$
$R_{i}$	Payment given to worker $w_{i}$
$will_{i}$	Willingness of worker $w_{i}$
$WF_{ij}$	Willingness factor of worker $w_{i}$ for task $t_{j}$
$Wrt_{ij}$	Response time of worker $w_{i}$ for task $t_{j}$
$P_{i}^{r}$	Residual power of worker $w_{i}$
$W_{T_{i}}$	Task $t_{j}$ popularity
$SP_{ij}$	Specification of worker $w_{i}$ for task $t_{j}$
$SP_{iT_{t}^{w_{i}}}$	Specification of worker $w_{i}$ for $T_{t}^{w_{i}}$
$Pr_{i}$	Profile for worker *i*
**Task Parameter**	
*T*	Set of all tasks
*m*	Number of task
$will_{j}$	Willing threshold of task $t_{j}$
$t_{j}$	Task *j*
$te_{j}$	End time of task $t_{j}$
$ts_{j}$	Start time of task $t_{j}$
$t_{d_{j}}$	Sensing duration of task $t_{j}$
${bt}_{j}$	Budget allocated to task $t_{j}$
$Pr_{j}$	Profile for task $t_{j}$
$T_{t}^{w_{i}}$	Set of interested tasks for worker $w_{i}$
$TD_{j}$	Task Demand for task $t_{j}$
**Other**	
*B*	Total budget
$D(w_{i},t_{j})$	Distance between worker $w_{i}$ location and the task $t_{j}$ position

**Table 2:** Average performance w.r.t Varying Number of Tasks.

Workers’ Welfare Average Performance Based on the Number of Tasks			
	WMTA-GA	GWR-GA	GWP
WMTA-GA	-	2.91	73.24
GWR-GA	−3	-	68
GWP	42	−40.60	-

**Table 3:** Average performance w.r.t Varying Number of Workers.

Workers Welfare Average Performance Based on Number of Workers			
	WMTA-GA	GWR-GA	GWP
WMTA-GA	-	1.98	17.26
GWR-GA	−2	-	15
GWP	−15	−13.03	-

## Data Availability

Data sharing avaliable upon request.
